# Supplementary material for: Predictors of internalised stigma among people with mental illness attending a psychiatry outpatient clinic in Ethiopia: Institution based cross sectional study
Source: PLoS One. 2025 Mar 18;20(3):e0319458. doi: 10.1371/journal.pone.0319458 (PMC11918445; doi:10.1371/journal.pone.0319458)
Supplement: Attachment — Submitted filename: PONE-D-24-17598_comments.docx [file pone.0319458.s001.docx]

**Predictors of internalised stigma among people with mental illness attending a psychiatry outpatient clinic**

Overall, the manuscript provides valuable insights into the relationship between metacognitive beliefs, mood symptoms, and fatigue in stroke survivors. It is generally well-written and organized, with a clear introduction providing context for the study. However, addressing the recommendations would enhance the clarity, robustness, and applicability of the findings.

**General recommendation:**

1. The study's cross-sectional design limits its ability to establish causal relationships. The manuscript acknowledges this limitation, but it would be beneficial to explicitly discuss the implications of this design choice on the interpretation of results and suggest avenues for future longitudinal research.

**More specific recommendation:**

**Introduction**

The introduction provides a thorough overview of the stigma associated with mental illness, distinguishing between internalized and perceived stigma. It effectively highlights the public health significance of stigma and the need for research in low and middle-income countries, especially Ethiopia. The literature review is comprehensive, addressing various factors contributing to stigma and noting the global relevance. However, it could benefit from a more in-depth discussion of cultural differences in stigma experiences and the rationale for choosing Ethiopia as the study site.

**Methods**

The methods section is well-detailed, covering the study design, sampling, and data collection procedures. The use of standardized tools, such as the ISMI-9 for measuring stigma, adds rigor to the study. However, the inclusion of more information about the training provided to data collectors and steps to minimize bias would strengthen the methodological transparency. Additionally, while the sample size calculation is justified, a discussion of potential limitations related to excluding severely impaired individuals could provide a more balanced view.

- The title of “Tools used for data collection” should be renamed to “measurement”

**Results**

The results are presented clearly, with appropriate use of statistical analysis to identify significant predictors of internalized stigma. The prevalence data and associations with factors like education level, suicidal ideation, and substance use are reported effectively. However, some sections could be enhanced with visual aids, such as charts or graphs, to better illustrate the relationships among variables. Additionally, discussing the clinical implications of the findings, especially for healthcare providers in Ethiopia, would add practical value.

**Discussion**

The discussion section successfully interprets the results in the context of existing literature, noting consistencies and differences with previous studies. It provides plausible explanations for the observed associations and acknowledges cultural factors that may influence internalized stigma. However, the discussion could be enriched by addressing the implications for policy and service development, particularly regarding mental health education and support systems. The limitations are acknowledged, but a deeper exploration of potential confounding variables would strengthen this section.

**Conclusion**

The conclusion effectively summarizes the key findings and reinforces the need for anti-stigma efforts in Ethiopia. It provides clear recommendations, such as early outpatient support and drug adherence interventions, but could be further strengthened by suggesting specific anti-stigma strategies tailored to the Ethiopian cultural context. Additionally, highlighting areas for future research, such as longitudinal studies to explore causality, would offer a more forward-looking perspective.
