# Supplementary material for: Predictors of internalised stigma among people with mental illness attending a psychiatry outpatient clinic in Ethiopia: Institution based cross sectional study
Source: PLoS One. 2025 Mar 18;20(3):e0319458. doi: 10.1371/journal.pone.0319458 (PMC11918445; doi:10.1371/journal.pone.0319458)
Supplement: Attachment — Submitted filename: Response to reviewers.docx [file pone.0319458.s002.docx]

Point by point response for: **Predictors of Internalised Stigma Among People with Mental Illness Attending a Psychiatry Outpatient Clinic in Ethiopia: Institution Based Cross Sectional study.**

Manuscript Number PONE-D-24-17598

| **Editors’ comments** | **Author response** |
| --- | --- |
| 1. The manuscript contains important finding regarding the internalized stigma. However, please revise some comments raised by the reviewers giving more focus to the ethical issues, validity of the tools and editorial components. | Thank you for your constructive comments and suggestions to improve our manuscript and its acceptance for audience. We have addressed the comments and made relevant changes using tracked changes. The tool we used have good validity with Psychometric evaluation of the ISM-9 in the current sample showed high-scale reliability (Cronbach's alpha=0.88). We have included this information in the paper. We have also confirmed that informed consent was obtained from all participants and sought to address the editorial comments. |
| Reviewer-1 | Author response |
| 1. The study's cross-sectional design limits its ability to establish causal relationships. The manuscript acknowledges this limitation, but it would be beneficial to explicitly discuss the implications of this design choice on the interpretation of results and suggest avenues for future longitudinal research. | Thank you we have added further comment on this in the limitation section. |
| 2. The literature review is comprehensive, addressing various factors contributing to stigma and noting the global relevance. However, it could benefit from a more in-depth discussion of cultural differences in stigma experiences and the rationale for choosing Ethiopia as the study site. | Thank you. We have included the rationale for selecting Ethiopia as the study site in the introduction of the manuscript. |
| 3. The inclusion of more information about the training provided to data collectors and steps to minimize bias would strengthen the methodological transparency. | We have included further information about the training provided to data collectors. |
| 4. Additionally, while the sample size calculation is justified, a discussion of potential limitations related to excluding severely impaired individuals could provide a more balanced view. | Thank you, we have included the limitation of excluding severely ill individuals which cannot participate in the study because of the severity of the disease |
| 5. Tools used for data collection should be renamed to “measurement | Thank you, we have revised it on the document. We have also included further information on the tool here. |
| 6. Results - some sections could be enhanced with visual aids, such as charts or graphs, to better illustrate the relationships among variables. | Thank you. The magnitude of high internalised stigma and the types of mental illness are presented in a graph, while sociodemographic and other characteristics are displayed in tables, respectively. |
| 7. Discussing the clinical implications of the findings, especially for healthcare providers in Ethiopia, would add practical value. | Thank you. We have included in the discussion's final paragraph an explanation of how the implications of this finding are particularly relevant for Ethiopia and for healthcare providers working in this field. |
| 8. Discussion   The discussion could be enriched by addressing the implications for policy and service development, particularly regarding mental health education and support systems. | Thank you, Reviewer. We have presented our discussion in a comprehensive and logically organized manner, highlighting the significance of our findings and their broader relevance to the field of mental health and public health. Finally, we acknowledge the limitations of the study, particularly those that cannot be addressed due to the cross-sectional design. |
| 9. The limitations are acknowledged, but a deeper exploration of potential confounding variables would strengthen this section. | We have added further comment on confounding variables. |
| 10. Conclusion - The conclusion effectively summarizes the key findings and reinforces the need for anti-stigma efforts in Ethiopia. It provides clear recommendations, such as early outpatient support and drug adherence interventions, but could be further strengthened by suggesting specific anti-stigma strategies tailored to the Ethiopian cultural context. Additionally, highlighting areas for future research, such as longitudinal studies to explore causality, would offer a more forward-looking perspective. | Thank you, reviewer, we have revised as recommended |
| Reviewer #2: |  |
| 1. the authors did not obtain prior informed consent from the participants or their legal guardians before including them in the study. | Thank you, reviewer, we got written informed consent from participants prior to participation. Participants were fully briefed on the study's objectives and informed of their freedom to withdraw without any repercussions. We have included this in the paper. |
