# Supplementary material for: Predictors of internalised stigma among people with mental illness attending a psychiatry outpatient clinic in Ethiopia: Institution based cross sectional study
Source: PLoS One. 2025 Mar 18;20(3):e0319458. doi: 10.1371/journal.pone.0319458 (PMC11918445; doi:10.1371/journal.pone.0319458)
Supplement: Attachment — Submitted filename: pone.0319458.docx [file pone.0319458.s004.docx]

Point by point response for: **Predictors of Internalised Stigma Among People with Mental Illness Attending a Psychiatry Outpatient Clinic in Ethiopia: Institution Based Cross Sectional study.**

Manuscript Number PONE-D-24-17598

| **Editors’ comments** | **Author response** |
| --- | --- |
| 1. Review your reference list to ensure that it is complete and correct. | Thank you for your constructive comments and suggestions to improve our manuscript and its acceptance by the audience. We have addressed your feedback and made relevant changes, which can be viewed using tracked changes. Regarding the reference list, it is complete. Based on the information we have; we did not include or cite any papers that have been retracted. |
| Reviewer-2 |  |
| 1. Is the manuscript presented in an intelligible fashion and written in standard English? | Thank you for such encouraging feedback and kind words regarding our manuscript. We really appreciate the time and effort you have invested in reviewing our work and providing positive feedback. We have reviewed and proofread the document with the help of a native English speaker. |
| Reviewer-3 | Author response |
| 1. Reviewer #3: In the statistical analysis, it is better to add units of measurement on Age. | Thank you for this feedback. We have added the unit of measurement for age in the revised version of the manuscript. |
| 2. Regarding ethical standard as the study involves human subjects, it is better to provide ethical statement. And on submission guidelines, the abstract should be within the limited range, it shouldn't beyond 300 words. | Thank you again for these suggestions. Regarding the inclusion of ethical considerations. We have included the ethical statement and ethics approval in the methods section and the submission guidelines for ethics approval. Additionally, we have revised the abstract to be below the 300-word limit, in accordance with recommendations and journal requirements. |
